# The Moderating Effects of the Families Improving Together (FIT) for Weight Loss Intervention and Parenting Factors on Family Mealtime in Overweight and Obese African American Adolescents

**DOI:** 10.3390/nu13061745

**Published:** 2021-05-21

**Authors:** Dawn K. Wilson, Allison M. Sweeney, Mary Quattlebaum, Haylee Loncar, Colby Kipp, Asia Brown

**Affiliations:** 1 Department of Psychology, University of South Carolina, Columbia, SC 29208, USA; mjq@email.sc.edu (M.Q.); gauseh@email.sc.edu (H.L.); ckipp@email.sc.edu (C.K.); asia@email.sc.edu (A.B.); 2 College of Nursing, University of South Carolina, Columbia, SC 29208, USA; sweeneam@mailbox.sc.edu

**Keywords:** parenting factors, intervention, family mealtime, authoritative, African American

## Abstract

Few studies have integrated positive parenting and motivational strategies to address dietary outcomes such as frequency of family mealtime. The Families Improving Together (FIT) for Weight Loss trial was a randomized group cohort trial (*n* = 241 dyads) testing the efficacy of integrating a motivational plus family weight loss (M + FWL) intervention for healthy eating and weight loss in overweight and obese African American adolescents. The current study tested the interaction of parenting styles (responsiveness, demandingness) and parental feeding practices (restriction, concern about child’s weight, pressure to eat) and the FIT intervention on frequency of family mealtime over 16 weeks. Multilevel modeling demonstrated significant interactions between the group-based treatment and responsiveness (*p* = 0.018) and demandingness (*p* = 0.010) on family mealtime. For the group-based M + FWL intervention, increased responsiveness and reduced demandingness were associated with increased frequency of family mealtime from baseline to 16 weeks. There was also a negative association between parental restriction and frequency of family mealtime, but a positive association between parental concerns about their adolescent’s weight and frequency of mealtime. These findings are the first to demonstrate that an authoritative or nurturing parenting style moderated intervention effects for improving the frequency of family mealtime in overweight and obese African American adolescents.

## 1. Introduction

The prevalence of obesity among African American adolescents is a major public health concern, with approximately 40% overweight or obese [1,2]. Obesity in adolescence often persists into adulthood, and is accompanied by detrimental physical, psychological, and social consequences [3]. African American adolescents may have a greater risk of living in obesogenic environments, thus contributing to differences in prevalence rates of obesity [4]. Numerous studies have shown that family-related factors, including more frequent family meals, are associated with improved dietary intake and family functioning among adolescents [5,6,7]. The current study tested whether parenting styles (responsiveness, demandingness) and parental feeding practices (restriction, concern about child’s weight, pressure to eat) moderated the effect of the Families Improving Together (FIT) for Weight Loss Intervention on frequency of family mealtime in African American families with overweight and obese adolescents.

Family Systems Theory (FST) proposes that supportive, nurturing family interactions and positive parenting behaviors (warmth, communication) are important for promoting healthy development in adolescence, such as nutritious eating behaviors [8,9,10]. Parenting styles, including authoritative (high responsiveness, high demandingness) and authoritarian (low responsiveness, high demandingness), have shown important associations with adolescents’ eating behaviors and dietary intake [11]. Specifically, authoritative practices have been linked to higher fruit and vegetable intake (F&V) in children and adolescents [12], whereas authoritarian practices have been related to lower F&V intake [13]. In line with FST, parental feeding practices such as more restrictive parental feeding practices, including restricting youths’ access to foods, pressuring youth to eat certain foods, and parental concerns about their youths’ weight have been linked to poorer-quality dietary intake [14,15]. Furthermore, parental styles such as responsiveness (i.e., high nurturance), may help facilitate a supportive home climate, which may moderate the effects of adolescent dietary patterns to improve outcomes such as family mealtime [10].

The FIT trial was developed to promote positive weight-related behavioral changes in overweight African American adolescents by encouraging a supportive family climate, positive parent–child communication, and parental autonomy support [16]. Previous research has shown that providing autonomy support is an important skill for parents as adolescents engage in increasingly independent decision making into young adulthood [17]. Engaging in shared decision making (i.e., allowing for input, offering choices) may also increase motivation and adolescent engagement in positive health behaviors [17]. For example, a previous study showed positive associations between parental autonomy support and adolescent intrinsic motivation and improved adolescent health behaviors and adherence to weight loss treatment [18]. Berge et al. [7] also found higher levels of family functioning (communication, joint problem solving, closeness) were related to healthier body mass index (BMI), dietary intake, and physical activity in adolescents. Similar results were found by Haines et al. [19] who showed significant relationships between higher family functioning and lower obesity risk among adolescents.

Few previous studies have examined the moderating effects of parenting styles and parental feeding practices on dietary behaviors related to family mealtime in overweight African American adolescents. One study, however, implemented positive parenting strategies to promote healthy eating and physical activity known as the Group Lifestyle Triple P trial [20]. This intervention used a multilevel system to improve parenting, and family support aimed at preventing social, emotional and behavioral problems in youth. The study showed significant decreases in child weight-related outcomes and parents reported increases in their confidence in managing their child’s weight-related issues. Another more recent study showed that parental warmth at baseline was associated with improved weight-related maintenance during a standard family-based behavioral weight control program [21]. Taken together, these studies provide support for the association between positive parenting and childhood health behaviors related to obesity prevention. Little research has focused, however, on family mealtime as a critical outcome variable of interest. Past studies have shown that more frequent family meals are related to lower risk for overweight and healthier diet in adolescents [22]. Other studies, however, have found no association between these variables, highlighting the need for continued research [23,24]. Berge and colleagues [7] did report that general family functioning was associated with more frequent family meals but did not evaluate whether parenting styles or parental feeding practices were critical mechanisms. Thus, further research is needed to better understand the relationship between parenting styles and parental feeding practices (e.g., responsiveness, demandingness, restriction, concern) and weight loss treatments on frequency of family mealtime especially among underserved, low-income African American youth who are at increased risk for obesity and related chronic diseases.

The purpose of the current study was to examine whether parenting styles (responsiveness, demandingness) and parental feeding practices (restriction, concern about adolescent weight, pressure to eat) moderated the effects of the FIT intervention on frequency of family mealtime from baseline to 16 weeks. Based on FST and previous research [8], it was hypothesized that parenting responsiveness and demandingness would moderate the treatment effect of the FIT intervention, such that increases in responsiveness and decreases in demandingness would be associated with increased family mealtime in the FIT intervention. Additionally, it was hypothesized that decreases in restriction and pressure to eat and increases in concern would be associated with increased family mealtime in the FIT intervention but not the control group condition.

## 2. Materials and Methods

### 2.1. Participants

A total of 241 families (dyad of parent/caregiver and adolescent) participated in the FIT trial. Families were recruited through community partnerships with local churches, pediatric clinics, and schools, as well as by culturally relevant ads, community events, and word of mouth [25]. Families were considered eligible if: (1) they had an African American adolescent between the ages of 11 and 16 years old, (2) the adolescent was overweight or obese, defined as having a ≥85th BMI percentile for age and sex, (3) at least one parent or caregiver living in the household was willing to participate, and (4) the family had internet access. Exclusion criteria included presence of a medical or psychiatric condition that would interfere with dietary behaviors or physical activity, they were already participating in a weight loss program or taking medication that could interfere with weight loss. If the family had more than one eligible adolescent, one was selected at random to complete measures, and the entire family was invited to participate in the group sessions. The trial was approved by the University of South Carolina Institutional Review Board and was registered with ClinicalTrials.gov (NCT#01796067). All parents signed informed consent for them and their adolescent and were told they could discontinue the program at any time. Each family was compensated a total of $110 for their participation in the FIT trial distributed across measurement time points.

### 2.2. FIT Trial Study Design

The FIT trial was a group cohort randomized controlled trial, which was designed to test the efficacy of a motivational plus family weight loss program (M + FWL) versus a comprehensive health education (CHE) control group [16,26,27,28]. The study utilized a 2 × 2 factorial design to test the effects of a group-based face-to-face program (M + FWL vs. CHE) and the added dose effects of a tailored online program versus control online program. Families first participated in a 2 week orientation phase which allowed participants to learn more about the program, complete baseline measures, and identify barriers to participation. Families who successfully completed the run-in orientation phase were randomized to the first phase of the trial which tested the efficacy of a group-based 8 week face-to-face program (M + FML vs. CHE). Families were then re-randomized for phase 2 of the trial which compared an 8 week tailored online program to a control online program. Following the online program, families received 3 online booster sessions (1 every 2 months), which corresponded to the online program to which they were randomized. Measures were collected at baseline, after the face-to-face program (8 weeks), after the online program (16 weeks), and at 6 months post-intervention. The current study examined baseline to 16 weeks post-intervention.

### 2.3. FIT Motivational and Family Weight Loss (M + FWL) Intervention

The curriculum for the FIT M + FWL intervention integrated elements from Social Cognitive Theory [29], Self-Determination Theory [17], and Family Systems Theory [8], as well as cultural tailoring strategies to target weight-related outcomes in African American youth. Essential elements included autonomy, parent social support, communication skills, parental monitoring, goal setting, self-monitoring, and behavioral skills (see also [28] for full details). The FIT trial used these components to specifically target: (1) increasing F&V intake, (2) decreasing fast food and junk food intake, (3) decreasing sugar-sweetened beverages, (4) increasing physical activity, and (5) decreasing screen time (see [16] for full description of the FIT intervention).

The face-to-face component of the FIT intervention was delivered by two facilitators (at least one of whom was African American) who received training on behavioral skills, positive parenting, motivational interviewing, and cultural competency. During each of the 8 weeks, 5–10 families meet for 1.5 h with the facilitators in groups to discuss topics including positive parenting and communication skills, self-monitoring and goal setting, energy balance and portion sizes, physical activity and sedentary behavior, and relapse prevention. Parents and adolescents received personalized daily calorie goals designed to promote gradual weight loss early in the program. A supportive, interactive group environment was emphasized, and facilitators modeled providing choices and supportive encouragement around setting health goals. Parents were encouraged to provide choice to their child and engage in shared decision making. At the end of each session, family bonding activities were recommended, which were designed to encourage positive parenting skills and reinforce behavioral changes. Families also received individualized feedback for approximately 15 min each week that included a self-assessment of diet and physical activity behaviors, discussion of self-monitoring logs with the facilitator, and problem solving and goal setting for the next week. Make-up sessions were available in person or by phone [27].

Both caregivers and adolescents completed a tailoring survey after the face-to-face phase of the intervention, which was used to tailor the FIT online intervention. The FIT online intervention was completed by the parent/caregiver and was designed to encourage positive parenting skills to support adolescents’ weight loss goals. The online program consisted of 8 weekly online sessions and 3 booster sessions (1 every 2 months), which could be completed whenever was convenient for the participant during each week. Participants were reminded each week to log on to complete the program by a research assistant who was available for technical assistance. The online program was tailored on the following elements: cultural factors, personal values, motivation, parent communication style, and current and past health behaviors that the adolescent was willing to work on. Parents completed a survey each week describing their adolescent’s progress and received tailored feedback. Participants were able to select from the following content areas each week which targeted a health behavior that was paired with a parenting strategy: (1) energy balance and meeting a calorie goal/active listening, (2) fast food/reverse role play, (3) fruits and vegetables/increasing engagement, (4) physical activity/escape hatch, volition, choice, (5) time spent sitting/you provide, they decide, and (6) sweetened beverages/push versus pull. Choices were presented in order of adolescent behavior and willingness to change (with behaviors the adolescent was willing to change presented first). Parents set an action plan at the end of each session.

### 2.4. Comprehensive Health Education (CHE) Comparison Program

Groups sessions for the CHE program also took place for 1.5 h weekly for 8 weeks and covered topics including stress management, diabetes, hypertension, cancer, media literacy, metabolism, positive self-concept, and sleep. The CHE curriculum did not include parenting skills or behavioral components. Online sessions for the 8 week control program were also completed by the parent/caregiver and included tobacco prevention, social media and parenting, bullying and peer relationships, oral hygiene, nutrition, depression, sleep, and family stress. Topics were presented in order and were not tailored.

### 2.5. Measures

#### 2.5.1. Demographic Information

Demographic information was assessed at baseline and included adolescent sex, parent sex, parent age, parent marital status, number of children in the household, parent education, and annual household income was self-reported by the parent at baseline. Adolescent age was calculated at the time of baseline measurement using the birth date of the adolescent and the date of the measurement appointment.

#### 2.5.2. Adolescent and Parent BMI

Measures of weight and height were obtained by trained staff in order to objectively calculate adolescent and parent BMI. Specifically, two measures of weight and height were assessed for each participant using a Seca 880 digital scale and a Shorr height board, respectively. The two height and weight measurements were averaged in order to minimize error, and BMI was calculated using these averages. Adolescent BMI (zBMI) was standardized for age and biological sex using the Centers for Disease Control growth curves [30].

#### 2.5.3. Adolescent Perceptions of Parenting Style (Responsiveness and Demandingness)

The Authoritative Parenting Index was used to assess parental responsiveness and demandingness which is a six items measure of the adolescent’s perspective [31]. Responses were scored on a 5-point Likert scale ranging from 1 “not at all like me” to 5 “exactly like me.” Sample items included “My parents make me feel better when I am upset” (responsiveness) and “My parents have rules that I must follow” (demandingness).

These subscales have also been validated for African American samples [31] and shown to have construct validity [31,32]. Additionally, these subscales for demandingness and responsiveness have been shown to be reliable for adolescents in the present study (α = 0.65 and 0.78, respectively).

#### 2.5.4. Adolescent-Reported Parental Feeding Practices

The Child Feeding Questionnaire (CFQ) was used to assess adolescents’ report of their parents’ feeding practices [33]. Items on this questionnaire were modified to reflect the adolescent’s perspective of parental feeding practices that has been validated in a prior study [34]. The present study includes items from three of the five CFQ subscales: parental restriction, parental concern about child’s weight, and parental pressure to eat. Adolescents reported their responses on a 5-point Likert scale to rate their level of agreement related to their parents’ feeding practices (“disagree” to “agree”) or how frequently their parents’ engaged in a feeding practice (“never” to “always”) for each subscale. Sample items for each subscale utilized in the present study included restriction (e.g., “My parent has to watch that I do not eat too much of my favorite food”), concern about child’s weight (e.g., “My parent is concerned about me eating too much”), and pressure to eat (e.g., “If I say ‘I’m not hungry,’ my parent tries to get me to eat anyway”). The questionnaire has been validated with adolescent samples, and the subscales utilized in the present study has shown to have adequate reliabilities (restriction: α = 0.72; concern: α = 0.82; pressure to eat: α = 0.71) [35].

### 2.6. Outcome Measures

#### Family Mealtime

Adolescents were asked the frequency of their family mealtimes during a typical week using a validated scale [36]. The response options were (never), 2 (1–2 times), 3 (3–4 times), 4 (5–6 times), 5 (7 times), and 6 (more than 7 times). This scale has also been used in diverse racial populations and shown to have construct validity [36].

### 2.7. Analysis Plan

Multilevel modeling was used to allow for the estimation of effects at multiple time points (baseline and 16 weeks) within adolescents. Measures of parenting style, parental feeding practices, and family mealtime were collected at baseline and 16 weeks, which allowed for the testing of whether changes in parenting style and factors predicted changes in family mealtime frequency, and whether these differences were greater in the intervention than control. Given that families were treated within treatment groups during the face-to-face program, random intercepts and random slopes for group were included in each model. Assumptions for multilevel modeling were evaluated, including the presence of influential cases, normality, and heteroscedasticity. Time was coded such that baseline = 0 and 16 weeks = 1. The group and online treatment were coded such that 1 = M + FWL intervention, 0 = CHE control. Adolescent age was mean centered, while income (1 = high income, ≥40 k per year, 0 = low income) and adolescent sex was dummy coded (1 = male, 0 = female). The parenting style variables (demandingness and responsiveness subscales) and parental feeding practices (concern, restrict, pressure to eat) were calculated by norming each item before summation to allow each item to contribute equally to the overall scale score (baseline and 16 weeks). Summed scale scores were transformed to z scores to aid in analysis and interpretation of statistical models. The model tested a series of three-way interactions to evaluate whether changes in parenting style and parental feeding practices (in the treatment vs. control) were associated with changes in the frequency of family meals from baseline to 16 weeks.

Psychosocial data, including measures of family mealtime, were missing from <1% of adolescents at baseline and 36.6% at 16 weeks. Multiple imputation was used to address missing data [37], which provides an unbiased estimate of parameters and standard errors and is appropriate for longitudinal trials [38]. All demographic data, primary and secondary outcomes from the FIT trial, and variables of theoretical importance, including family mealtime and parenting style and parental feeding practices, were included in the imputation in order to minimize the likelihood of biased estimates and meet missing at random assumptions. This process resulted in 20 imputed datasets. After generating the datasets, the proposed models were conducted using each dataset, with the final parameter estimates being pooled across imputations.

## 3. Results

### 3.1. Participant Characteristics

Demographic and baseline characteristics of the sample are presented in Table 1. The sample (*n* = 241) consisted of African American adolescents, with an average age of 12.8 (*SD* = 1.75) and an average BMI percentile of 96.63. The majority of parents were not married (65%), earned less than $40,000 annually (63%), and had completed some college or less (57.7%). A total of 75.5% of families completed the intervention program at 16 weeks.

### 3.2. Bivariate Correlations

Table 2 provides a correlation matrix of associations between the predictors and outcome variables. At baseline, frequency of family mealtime was low to moderately correlated with parental responsiveness (*r* = 0.15, *p* < 0.05) demandingness (*r* = 0.24, *p* < 0.05) and restriction (*r* = 0.24, *p* < 0.05). At baseline, responsiveness and demandingness were moderately correlated (*r* = 0.48, *p* < 0.05) and parent feeding practices had modest positive associations ranging from *r* = 0.15 to *r* = 0.41.

### 3.3. Parenting Style

**Figure 1:**
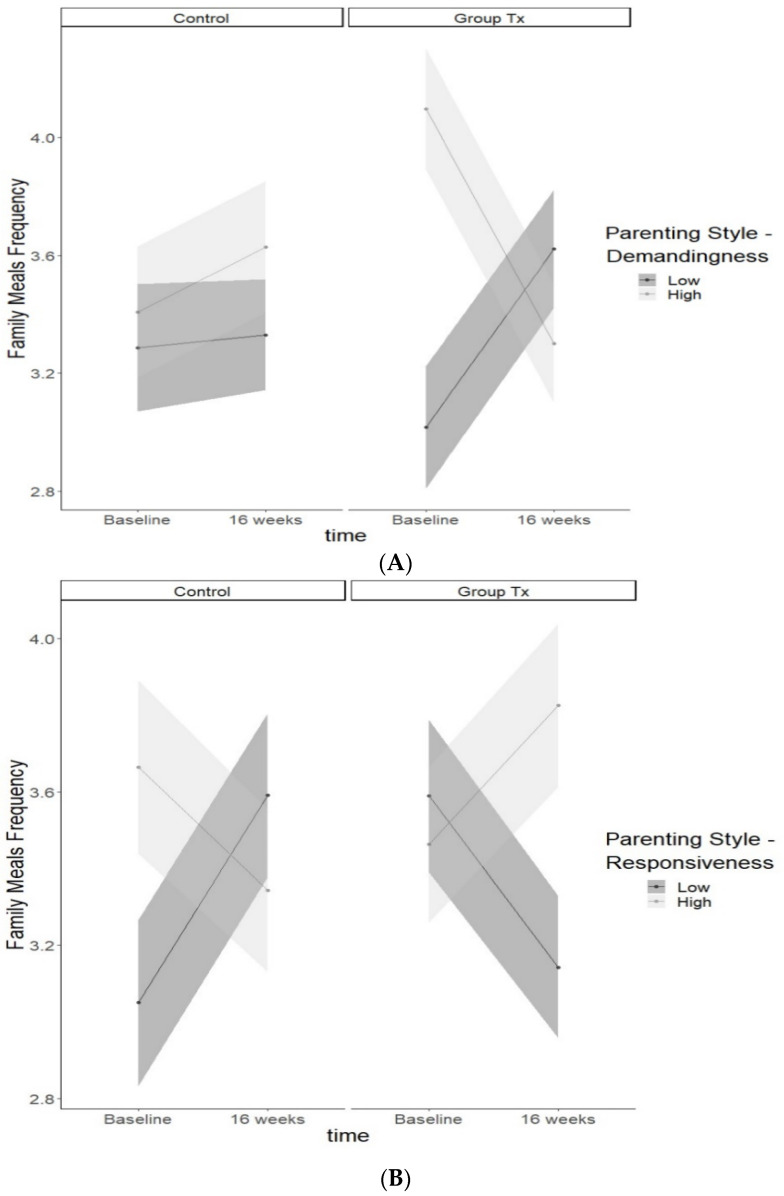
Three-way interaction between time x group treatment x demandingness (**A**) and time x group treatment x responsiveness (**B**) predicting frequency of family meals.

As shown in Table 3, a multilevel model was conducted to evaluate interactions between time, treatment (group and online), and parenting style (responsiveness, demandingness) on frequency of family meals at 16 weeks. There was a significant main effect for adolescent age, such that greater adolescent age was associated with lower frequency of family meals (estimate = −0.137, SE = 0.045, *p* = 0.002). Furthermore, there were two significant three-way interactions, including time x group treatment x responsiveness (estimate = 0.852, SE = 0.358, *p* = 0.018) and time x group treatment x demandingness (estimate = −0.947, SE = 0.364, *p* = 0.010). As shown in Figure 1, in the group treatment, greater demandingness was associated with a decrease in the frequency of family meals from baseline to 16 weeks, whereas lower demandingness was associated with an increase in the frequency of family meals from baseline to 16 weeks. Alternatively, in the CHE control group, both greater and lower demandingness were positively associated with the frequency of family meals from baseline to 16 weeks. Regarding responsiveness, in the group treatment greater responsiveness was associated with an increase in the frequency of family meals from baseline to 16 weeks, whereas lower responsiveness was associated with a decrease in the frequency of family meals from baseline to 16 weeks. In the CHE control group, greater responsiveness was associated with a decrease in the frequency of family mealtime from baseline to 16 weeks, whereas lower responsiveness was associated with an increase in the frequency of family meals from baseline to 16 weeks. There were no interactions between parenting style and online treatment.

### 3.4. Parenting Feeding Practices

**Figure 2:**
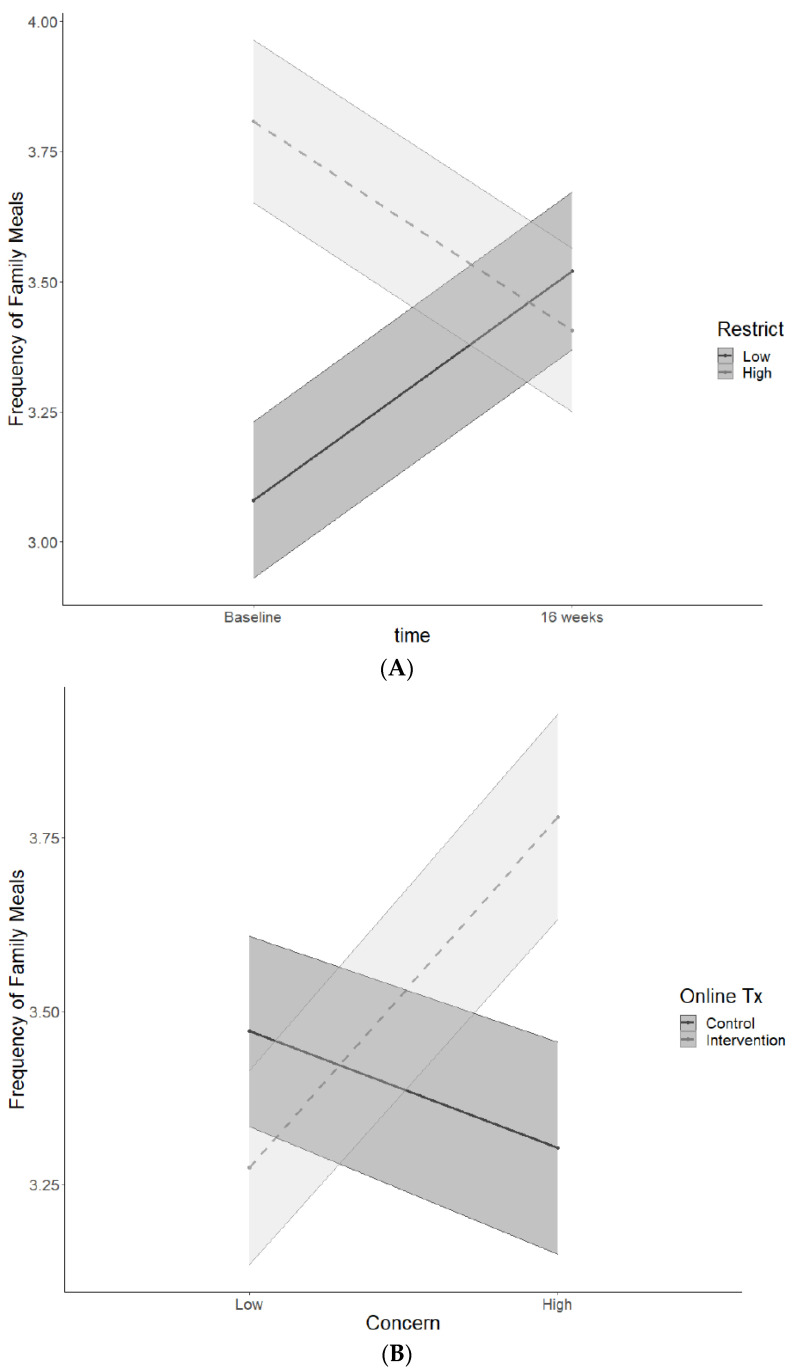
Two-way interactions with parenting feeding practices, including time x restriction (**A**) and online treatment x concern (**B**) predicting frequency of family meals.

A second multilevel model was conducted to evaluate interactions between time, treatment, and parental feeding practices (concern, restrict, pressure to eat) on frequency of family meals at 16 weeks (see Table 4). Again, there was a significant main effect for adolescent age (estimate = −0.123, SE = 0.047, *p* = 0.009). There were two significant two-way interactions, including time x restrict (estimate = −0.680, SE = 0.332, *p* = 0.042) and online treatment x concern (estimate = 0.542, SE = 0.223, *p* = 0.015). As shown in Figure 2, the time x restrict interaction revealed that lower restriction was associated with an increase in the frequency of family meals from baseline to 16 weeks, whereas higher restriction was associated with a decrease in family meals. This was not moderated by treatment, suggesting that restriction was associated with decreased frequency of family mealtime for both families in the intervention and control. Furthermore, the online treatment x concern interaction revealed that in the online treatment higher parental concern about an adolescent’s weight was associated with an increase in the frequency of family mealtime, whereas in the online control concern was associated with a decrease in the frequency of family mealtime (see Figure 2). This two-way interaction was not moderated by time, indicating that the association between concern and family mealtime was stable across time. There were no significant interactions or main effects with pressure to eat and no group-based treatment effects.

## 4. Discussion

The present study evaluated whether parenting style and parental feedback practices moderated the FIT treatment effects on frequency of family mealtime from baseline to 16 weeks. Both demandingness and responsiveness moderated the effect of the group treatment on family meals at 16 weeks. As hypothesized, for those in the group intervention condition, decreases in parental demandingness and increases in parental responsiveness were associated with a greater frequency of family mealtime. Conversely, for the group CHE control, the findings for parental responsivity on family mealtime were in the opposite direction as compared to the intervention group. There was also a negative association between parental restriction and frequency of family mealtime across time, and a positive association between parental concern and frequency of family meals in the online M + FWL intervention, but not for the online control group. These findings are the first to demonstrate that a more authoritative or nurturing parenting style (i.e., increases in responsiveness and decreases in demandingness) moderated the intervention effect of a family-based weight loss treatment for improving the frequency of family mealtime in African American families with overweight and obese adolescents.

An important finding in this study was that increases in parental responsiveness and decreases in demandingness moderated the FIT intervention effects on increasing the frequency of family mealtime. These findings are consistent with the findings on weight-related benefits of autonomy-supportive parenting across predominantly European American populations [39] as well as recent studies in overweight African American adolescents [40]. This finding is also consistent with other studies that have shown that parental warmth at baseline was associated with improved weight-related maintenance during a standard family-based behavioral weight control program with primarily White youth [21]. In addition, other studies have shown that specific parent behaviors such as the use of praise during family-based interventions have been associated with a decrease in child weight-related outcomes [41,42]. Parents who have an authoritative parenting style (high levels of warmth and support) have also been shown to have children who are more likely to engage in their own self-care behaviors around diabetes management [43] and have greater psychosocial functioning [44,45]. More supportive parenting may help adolescents’ confidence and self-regulation for making the required behavioral changes for engaging in healthy behaviors and promoting long-term engagement in new health behaviors [21]. Further research is needed to better understand what specific components of parenting and related family-based interventions are most important for sustaining family mealtime and family lifestyle changes over time.

Of note, the relationship between parental responsiveness and family mealtime was opposite of the group-based FIT intervention for the CHE control group, such that decreased responsiveness was associated with greater family mealtime. Further research is needed to better understand whether authoritarian parenting (low nurturance, high control/demandingness) may be beneficial in some circumstances for overweight African American adolescents’ overall wellbeing. For example, previous research has shown that authoritarian parenting can increase assertiveness and independence in adolescents, especially in populations with low income [46]. However, very little past research has examined how parenting practices impact overall eating and family mealtime in an entirely African American adolescent sample [7,47,48,49]. Some studies have suggested that parenting may function differently in African American families than European American families. These studies have shown that authoritarian parenting is associated with more positive outcomes, such as independence, maturity and self-regulation for African American as compared to European American youth [50,51,52]. Thus, further research is needed to better understand the cultural values underlying parenting practices and especially more restrictive practices and under what conditions these parenting styles may be useful versus harmful.

A novel contribution of the current study is its assessment of parental feeding practices in an African American adolescent population. This study is one of the few studies to assess the moderating relationship between parenting factors and an intervention on frequency of family mealtime in overweight and obese African American adolescents [36,53]. Parent restriction of diet was associated with lower frequency of family mealtime across time, which was not moderated by treatment, suggesting that increases in restriction across time had a negative impact on family mealtime regardless of treatment allocation. Additionally, parental concern about adolescent weight status was associated with greater frequency of family mealtime in the online treatment but not the online control group. These are important findings given that some studies have found that families with regular family meals have been shown to have higher positive family functioning [7]. However, one study showed that the effects of low family functioning among females but not males resulted in unhealthy dietary behaviors such as eating disorders [54]. Thus, further research is needed to better understand how parental restriction and concerns about weight link to the frequency of family mealtimes in overweight and obese adolescents.

This study has several limitations that should be considered when interpreting the results. This study incorporated a longitudinal design and used baseline psychosocial surveys of parenting factors and adolescent assessments of frequency of family mealtime gathered at baseline and 16 weeks. Future research is needed to better examine further how changes in parenting impact family mealtime and family routines over longer periods of time. Items in the present study were modified to reflect the adolescent’s perspective of parental feeding practices, but other investigators have validated this modified approach in previous studies [34,40]. This study is among the first to assess these moderating effects in an entirely African American adolescent sample, but generalizability of the findings may be limited and further studies are needed to replicate these findings. The sample has limited variability, as it included a small sample of overweight African American youth in the Southern United States, limiting applicability to families of different racial/ethnic backgrounds or families with normal-weight adolescents.

## 5. Conclusions

In conclusion, the findings from this study are the first to demonstrate that a more authoritative or nurturing parenting moderated intervention effects for improving the frequency of family mealtime in African American families with overweight and obese adolescents. Future intervention studies are needed to better understand how cultural values and parenting styles such as responsive parenting may be most useful in overweight and obese ethnic/minority adolescents and their families.

## Figures and Tables

**Table 1:** Descriptive data for the total sample at baseline (*n* = 241).

Adolescent sex (*n*, % female)	153 (63.5%)
Adolescent Age (years) *M* (*SD*)	12.8 (1.75)
Average Adolescent BMI %	96.63%
Parent sex (*n*, % female)	231 (96%)
Parent Age (years) (*M*, *SD*)	43.2 (8.65)
Parent BMI (kg/m^2^) (*M*, *SD*)	37.75 (8.79)
Married (*n*, %)	83 (34.4%)
Parent Education (*n,* %)	
9 To 11 Years	6 (2.5%)
12 Years	33 (13.7%)
Some College	100 (41.5%)
4 Year College	47 (19.5%)
Professional	55 (22.8%)
Parent Annual Household Income (*n*, %)	
Less than $10,000	36 (14.9%)
$10,000–$24,000	50 (20.3%)
$25,000–$39,000	66 (27.4%)
$40,000–$54,000	32 (13.3%)
$55,000–$69,000	21 (8.7%)
$70,000–$84,000	12 (5%)
$85,000 or greater	24 (10%)
Frequency of Family Meals-baseline, *M*(*SD*)	3.46 (1.62)

**Table 2:** Bivariate correlations between predictors and family mealtime.

	1	2	3	4	5	6	7	8	9	10	11	12	13	14
1. Adolescent Age	−	−0.12	−0.22 *	−0.06	−0.03	−0.07	−0.21 *	−0.12	−0.08	0.02	−0.06	−0.12	−0.20 *	−0.15 *
2. Adolescent zBMI		−	0.08	−0.08	−0.03	0.17 *	0.12	0.02	−0.03	−0.05	0.04	0.04	−0.06	−0.14 *
3. Family Meals (BL)			−	0.15 *	0.24 *	0.12	0.24 *	0.05	0.26 *	0.03	0.14 *	0.09	0.08	0.02
4. Responsiveness (BL)				−	0.48 *	0.28 *	0.22 *	0.01	0.12	0.38 *	0.16 *	0.13 *	−0.03	0
5. Demandingness (BL)					−	0.29 *	0.19 *	0.1	0.05	0.2 *	0.3 *	0.16 *	−0.03	0.07
6. Concern (BL)						−	0.36 *	0.15 *	0.02	0.08	0.13 *	0.36 *	0.06	0.14 *
7. Restrict (BL)							−	0.41 *	0.07	0.06	0.08	0.25 *	0.22 *	0.11
8. Pressure (BL)								−	0.03	−0.05	−0.14 *	0.01	0.11	0.24 *
9. Family Meals (16 w)									−	0.06	−0.08	0.05	−0.02	−0.08
10. Responsiveness (16 w)										−	0.47 *	0.19 *	0	−0.09
11. Demandingness (16 w)											−	0.31 *	0.09	−0.01
12. Concern (16 w)												−	0.3 *	0.22 *
13. Restrict (16 w)													−	0.47 *
14. Pressure (16 w)														−

Note. * = *p* < 0.05; BL = baseline.

**Table 3:** Multilevel model with parenting style predicting family mealtime from baseline to 16 weeks.

	*Estimate*	*SE*	*p*	*FMI*
Fixed Effects				
Intercept	4.849	0.697	<0.001	0.118
Time	−0.097	0.277	0.726	0.212
Group Treatment (tx)	0.189	0.200	0.344	0.001
Online Treatment (tx)	−0.019	0.199	0.922	0.001
Adolescent Age	−0.137	0.045	0.002	0.175
Adolescent Sex	0.075	0.157	0.634	0.113
Adolescent zBMI	0.115	0.157	0.462	0.160
Family income	0.047	0.160	0.771	0.148
Parenting Style—Responsiveness	0.289	0.220	0.188	0.002
Parenting Style—Demandingness	0.201	0.187	0.282	0.002
Time: Responsiveness	−0.530	0.321	0.099	0.131
Group tx: Responsiveness	−0.380	0.234	0.105	0.002
Time: Group tx	−0.143	0.316	0.651	0.190
Time: Demandingness	−0.068	0.307	0.824	0.222
Group tx: Demandingness	0.503	0.240	0.036	0.003
Online tx: Responsiveness	0.065	0.231	0.777	0.001
Time: Online tx	0.140	0.306	0.647	0.136
Online tx: Demandingness	−0.317	0.240	0.187	0.002
Time: Group tx: Responsiveness	0.852	0.358	0.018	0.196
Time: Group tx: Demandingness	−0.947	0.364	0.010	0.217
Time: Online tx: Responsiveness	0.056	0.345	0.871	0.159
Time: Online tx: Demandingness	0.405	0.351	0.249	0.164
Random Effects				
Intercept	0.000			
Group	0.000			
Residual	1.534			

**Table 4:** Multilevel model with parenting feeding practices predicting family mealtime from baseline to 16 weeks.

	*Estimate*	*SE*	*p*	*FMI*
Fixed Effects				
Intercept	4.853	0.732	<0.001	0.162
Time	−0.109	0.269	0.685	0.164
Group Treatment (tx)	0.097	0.204	0.636	0.002
Online Treatment (tx)	0.046	0.203	0.822	0.002
Adolescent Age	−0.123	0.047	0.009	0.203
Adolescent Sex	0.08	0.165	0.629	0.169
Adolescent zBMI	0.043	0.161	0.792	0.167
Family income	−0.032	0.159	0.84	0.118
Feeding Practice—Restrict	0.499	0.191	0.009	0.002
Feeding Practice—Concern	−0.168	0.199	0.400	0.007
Feeding Practice—Pressure to Eat	0.053	0.193	0.784	0.002
Time: Restrict	−0.68	0.332	0.042	0.307
Group tx: Restrict	0.004	0.242	0.986	0.001
Time: Group tx	−0.071	0.319	0.825	0.192
Online tx: Restrict	−0.284	0.241	0.239	0.00
Time: Online tx	0.075	0.306	0.807	0.12
Time: Concern	0.209	0.313	0.505	0.258
Group tx: Concern	−0.091	0.223	0.685	0.002
Online tx: Concern	0.542	0.223	0.015	0.001
Time: Pressure	−0.11	0.308	0.721	0.236
Group tx: Pressure	−0.122	0.221	0.583	0.001
Online tx: Pressure	−0.161	0.222	0.468	0.001
Time: Group tx: Restrict	0.248	0.367	0.500	0.151
Time: Online tx: Restrict	0.172	0.37	0.643	0.163
Time: Group tx: Concern	−0.131	0.33	0.692	0.141
Time: Online tx: Concern	−0.179	0.337	0.594	0.178
Time: Group tx: Pressure	0.06	0.373	0.872	0.289
Time: Online tx: Pressure	0.199	0.34	0.558	0.129
Random Effects				
Intercept	0.000			
Group	0.000			
Residual	1.540			

## Data Availability

The data presented in this study are available upon request from the corresponding author. The data are not currently publicly available, as the outcome paper has not yet been published.
